# Thiol Groups as a Biomarker for the Diagnosis and Prognosis of Prostate Cancer

**DOI:** 10.1038/s41598-020-65918-w

**Published:** 2020-06-04

**Authors:** Alexsandro Koike, Brunna Emanuella França Robles, Ana Gabriela da Silva Bonacini, Camila Cataldi de Alcantara, Edna Maria Vissoci Reiche, Isaias Dichi, Michael Maes, Rubens Cecchini, Andréa Name Colado Simão

**Affiliations:** 1 Cancer Institute of Londrina, Laboratory of Research in Applied Immunology, University of Londrina, Londrina, Paraná Brazil; 2 Laboratory of Research in Applied Immunology, University of Londrina, Londrina, Paraná Brazil; 3 Department of Pathology, Clinical Analysis and Toxicology, Laboratory of Research in Applied Immunology, University of Londrina, Londrina, Paraná Brazil; 4 Department of Internal Medicine, University of Londrina, Londrina, Paraná Brazil; 5 IMPACT Strategic Research Centre, School of Medicine, Deakin University, Geelong, Victoria Australia; 6 Department of Pathology Sciences, University of Londrina, Londrina, Paraná Brazil

**Keywords:** Inflammatory diseases, Prostate cancer

## Abstract

Oxidative stress (OS) is associated with the onset of prostate cancer (PCa). The aims of this study are to examine whether OS biomarkers may be employed as external validating criteria for the diagnosis PCa. This case-control study recruited 204 subjects, 73 patients with PCa, 67 patients with benign prostate hyperplasia (BPH), and 64 healthy controls (HC) and assayed plasma prostate-specific antigen (PSA), protein thiol (−SH) groups, lipid hydroperoxides, carbonyl proteins (PCB), advanced oxidation protein products (AOPP), and total radical-trapping antioxidant parameter (TRAP). -SH groups were significantly and inversely associated with PSA levels. PCa was characterized by lowered -SH groups and red blood cell TRAP levels, and higher PSA, AOPP and PCB levels as compared with BPH and HC. Support vector machine with 10-fold cross-validation showed that PSA values together with -SH groups, PCB and AOPP yielded a cross-validation accuracy of 96.34% for the differentiation of PCa from BPH and HC. The area under the ROC curve using PSA and -SH differentiating PCa from BPH and controls was 0.945. Moreover, lowered -SH, but not PSA, are associated with PCa metastasis and progression. Inflammatory biomarkers were not associated with PCa or BPH. PCa, its progression and metastatic PCa are characterized by lowered antioxidant defenses, especially lowered thiol groups, and increased oxidative stress toxicity, suggesting that these processes play a key role in the pathophysiology of PCa. An algorithm based on -SH and PSA values may be used to differentiate patients with PCa from those with BPH and controls.

## Introduction

PCa is the second most common malignancy in men and PCa and benign prostatic hyperplasia (BPH) are the most frequent prostatic diseases in aging men. Ageing is the major risk factor for PCa and BPH while also a positive familial history of PCa^1^, genetic factors^2,3^, ethnicity^4^, lifestyle and nutritional factors^5^, and obesity^6^ confer risk to PCa. Chronic prostatic inflammation is a possible risk factor that may be associated with PCa and BPH^7,8^. Metabolic Syndrome (MetS) may be associated with BPH, but not PCa^9^, and with the more aggressive PCa phenotypes^10^.

Oxidative stress (OS) toxicity is one of the mechanisms that is associated with the onset of BPH^11^ and PCa^12,13^ and progression of the latter. In addition, increased age is also associated with activated OS pathways^14^. Reactive oxygen species (ROS) are associated with carcinogenesis through structural DNA damage, interaction with oncogenes or tumor suppressor genes and/or immunological mechanisms^11,15^. In addition, ROS could be responsible for the formation of DNA adducts, for example with malondialdehyde (MDA), a byproduct of the peroxidation of polyunsaturated fatty acids. Oxidative lesions, which are not repaired, can lead to mutations increasing the risk of carcinogenesis^16^. Some studies found increased levels of MDA in patients with PCa and BPH as compared with controls^17,18^.

ROS-induced oxidative damage is exacerbated by a decreased efficiency of antioxidant defense mechanisms^16^. The level of one of these antioxidants namely total thiol (−SH) groups is inversely associated with aging and PCa progression. -SH concentrations are significantly lower in PCa patients while aging is associated with a moderate reduction of -SH in BPH patients^19^.

In the early stages of PCa, the differential diagnosis between PCa and BPH is not an easy task^20^. Increased total prostate-specific antigen (tPSA) is the most frequently used laboratorial biomarker to identify prostatic changes^21^, although tPSA has only a moderate sensitivity to discriminate between PCa and BPH as well as for predicting increased risk of metastasis^22^. Therefore, it is of paramount importance to delineate novel biomarkers, which could differentiate PCa from BPH and indolent prostate cancer from the more aggressive phenotypes. Moreover, the examination of oxidative stress pathways may delineate the mechanisms that contribute to the pathogenesis or pathophysiology of PCa thereby providing new drug targets to treat PCa. In addition, the side-effects of treatment of indolent tumors may cause increased morbidity as well as worsening of the quality of life without improving overall global survival, whilst treatment delay may lead to incurable disease^23^.

Thus, the main goal of this study is to identify biomarkers of OS that could differentiate BPH and PCa. The second goal is to delineate the OS biomarkers, which may be used in the clinical practice as predictors of PCa above and beyond the effects of PSA.

## Subjects and Methods

This is a case-control study that recruited 204 subjects, 73 patients with PCa (attending the Uro-Oncology Clinic of the Cancer Hospital, Londrina, Brazil), 67 patients with BPH (attending the Urology Clinic of the Evangelical Hospital of Londrina, Brazil), and 64 healthy volunteers (HC). Patients and controls were recruited from the same catchment area namely Londrina, Brazil. The age range of the participants was 35–69 years. The diagnosis PCa was made based on histological evaluation (positive biopsy results). Nine (12%) patients showed metastatic PCa. Moreover, the PCa group was subdivided according to the risk stratification National Comprehensive Cancer Network (NCCN) Guidelines^24^: AV group1: very low and low risk group; AV group2: favorable and unfavorable intermediate risk group; and AV group3: high and very high risk group. Exclusion criteria for normal controls included a prostate volume >30 cm^3^ (measured by transabdominal ultrasound) and tPSA >1.5 ng/mL, whereas BPH patients had prostate volume greater than 30 cm^3^. Exclusion criteria for patients and controls were: (a) treatments with anti-inflammatory drugs (except sporadic use of NSAIDs); (b) use of antioxidant supplements; (c) presence of immune-inflammatory disorders and acute or chronic infections; (d) a prior history of other cancers and; (e) chronic renal insufficiency.

MetS was defined following the Adult Treatment Panel III criteria namely three or more of the following five criteria are present: (1) waist circumference over 94 cm; (2) fasting triglyceride levels greater than or equal to 150 mg/dL; (3) high density lipoprotein (HDL) lower than 40 mg/dL; (4) blood pressure over 130/85 mmHg (or antihypertensive medication use) and; (5) fasting glucose levels greater than or equal to 100 mg/dL or the use of hypoglycemic medication^25^. Body weight was measured prior to blood collection, using electronic scales accurate to 0.1 kg, with patients wearing light clothing and no shoes; height was measured to a precision of 0.1 cm using a stadiometer. Body mass index (BMI) was calculated as weight (kg) divided by height (m) squared.

Written informed consent was given by all participants of this study. The Human Ethics Committee of the State University of Londrina approved the protocol (CAAE 56182916.7.0000.5231).

### Laboratorial analysis

Fasting blood was sampled in the morning hours (8.00 a.m.) and in PCa patients blood was sampled one week before surgery. EDTA collecting tubes with a standard anticoagulant concentration (0.5 mL of EDTA for each 4.5 mL of the blood sample) were used to collect samples for biochemical and OS biomarker assays. Samples were centrifuged at 804 g for 15 min and stored in plasma and serum aliquots at −70 °C until used in laboratory tests.

The assays of total PSA (tPSA), free PSA (fPSA), and ferritin were determined using a chemiluminescent microparticle immunoassay (CMIA; Architect, Abbott Laboratory, Abbott Park, IL, USA). Uric acid (UA) was evaluated by a biochemical auto-analyser (Dimension Dade AR, Dade Behring, Deerfield IL, USA). The quantification of -SH of proteins was performed with spectrophotometry, according to the method described by Hu^26^. This technique is based on the reaction of the 2,2-dithiobisnitrobenzoic acid (DTNB) with the thiol group of proteins as described by Reznick and Packer^27^ and the results were expressed in μM. Lipid hydroperoxides (LOOH) were evaluated as described by Flecha *et al*.^28^. The results were expressed in relative light units (RLU). Plasma protein carbonyls (PCB) were measured with a spectrophotometric method based on the reaction of 2,4-dinitrophenylhydrazine with the carbonyl group, forming 2,4-dinitrophenylhydrazone, according to^27^. The results were expressed in nmol/mg of proteins. AOPP was determined according to the method of Witko-Sarsat *et al*.^29^. The values were expressed in μM/L of T-chloramine equivalent. TRAP (total radical-trapping antioxidant parameter) was measured using the methodology described by Repetto *et al*.^30^. This method detects hydro and liposoluble antioxidants in the plasma and TRAP results are expressed in μM of Trolox. Serum levels of ferritin, fPSA and tPSA were determined using a chemiluminescent microparticle immunoassay (CMIA; Architect, Abbott Laboratory, Abbott Park, IL, USA). Serum levels of C-reactive protein (CRP) were determined by high sensitive turbidimetry (C8000, Abbott Laboratory, Abbott Park, IL, USA). The values of white blood cell (WBC) and hemoglobin (Hb) were obtained using a hematological equipment (BC-6800, Mindray, Shenzhen, China) and erythrocyte sedimentation rate (ESR) was performed using automated equipment (Alifax, Polverara, Itália).

### Statistics

Analysis of variance was used to assess between-group differences in scale variables and analysis of contingency tables (Χ2-test) was used to check associations between nominal variables. We used binary logistic regression analysis to delineate the most significant variables predicting PCa versus controls and or BPH while adjusting for possible confounders (including age, MetS, BMI). Multivariate general linear model (GLM) analysis was used to assess the effects of explanatory variables (including diagnosis) on the biomarkers while controlling for possible confounders. Tests for between-subject effects were used to assess the effects of significant explanatory variables on the separate biomarkers. Model-estimated estimated marginal mean (SE) values were calculated based on the multivariate GLM analysis and protected post hoc analyses were employed to check differences between PCa, BPH and HC. We p-corrected results of multiple comparisons for false discovery rate^31^. Receiver Operating Characteristics (ROC) analysis was used to compute the area under the ROC curve (AUC ROC). Statistical analyses were performed using IBM SPSS Windows versions 22 and 25. Tests were 2-tailed, and an alpha level of 0.05 indicated a statistically significant effect. Support Vector Machine (SVM) with radial basis kernel function was used to compute the correctly classified PCa and control cases using biomarkers as explanatory variables (The Unscrambler, CAMO) after 10-fold crosss-validation.

### Ethical approval

All procedures performed in studies involving human participants were in accordance with the ethical standards of the institutional and/or national research committee and with the 1964 Helsinki declaration and its later amendments or comparable ethical standards.

### Informed consent

All the participants included in this study provided written informed consent.

## Results

### Demographic data

Table 1 shows the demographic, clinical and biomarker data in HC, BPH and PCa. BPH and PCa patients were somewhat older than normal controls while there were no significant differences in familial PCa history, BMI, smoking rate, hypertension, and diabetes between the three groups. There were significantly more non-Caucasian people in the PCa group as compared with the BPH and control groups, while BPH was associated with a greater rate of MetS as compared with controls. This table also shows the measurements of PSA and OS biomarkers in the three study groups. Table 1 shows the results of analyses of variance with protected post-hoc comparisons but without any adjustment for the effects of extraneous variables. Therefore, we would suggest to focus on Tables 2 and 3 to interpret the adjusted biomarker data.

**Table 1:** Demographic, clinical and biomarker data in healthy controls (HC), patients with benign prostate hyperplasia (BPH) and prostate cancer (PCa).

Variables	HC^A^	BPH^B^	PCa^C^	F/X^2^	df	p
Age (years)	51.7 (8.6)^**B,C**^	63.3 (7.0)^**A**^	61.8 (7.4)^**A**^	44.9	2/201	<0.001
BMI (kg/m^2^)	28.4 (4.3)	27.2 (4.6)	27.3 (4.1)	1.60	2/200	0.205
Ethnicity (C/NC)	59/5^C^	66/1^**C**^	56/17^A,**B**^	17.71	2	<0.001
Smoking (N/Y)	54/10	56/11	65/8	1.01	2	0.605
MetS (N/Y)	36/28^**B**^	23/44^A^	36/37	6.66	2	0.036
Diabetes (N/Y)	59/5	54/13	62/11	3.67	2	0.159
Hypertension (N/Y)	40/24	32/35	42/31	3.01	2	0.222
PCa Familial Hx (N/Y)	33/31	31/36	26/47	3.70	2	0.157
tPSA (ng/mL)*	0.63 (0.42–0.95)^**B,C**^	2.24 (0.97–3.48) ^**A,C**^	6.87 (4.57–11.58)^**A,B**^	79.65	2/201	<0.001
fPSA (ng/mL)	0.22 (0.15)^**B,C**^	0.49 (0.39)^**A,C**^	1.24 (3.54)^**A,B**^	28.38	2/201	<0.001
LOOH_plasma_ (RLU)*	138.9 (19.5)	137.5 (21.3)	137.7 (24.7)	0.14	2/201	0.866
LOOH_RBC_ (RLU)*	264.0 (83.0)	293.5 (123.0)	280.7 (63.0)	1.60	2/201	0.204
AOPP (µmol/L of chloramines T equivalents)*	119.0 (54.8)^**B*,C***^	171.1 (100.2)^**A**^	175.9 (87.3)^**A**^	9.44	2/200	<0.001
PCB (nmol/mL/mg proteins)*	3.30 (1.01)	3.20 (0.99)^**C**^	3.67 (1.37)^**B**^	3.22	2/199	0.042
-SH groups (µM)*	372.8 (47.0)^**B,C**^	333.4 (47.8)^**A,C**^	251.1 (70.1)^**A,B**^	83.41	2/201	<0.001
Uric acid (mg/dL)*	5.87 (1.31)	5.67 (1.30)	5.63 (1.47)	0.57	2/201	0.566
TRAP_RBC_ (μM of Trolox)	1664 (279)	1618 (258)	1522 (313)	3.28	2/148	0.041
TRAP_plasma_ (μM of Trolox)	918.8 (156.9)	871.9 (122.5)	876.0 (137.4)	2.27	2/178	0.106

All values are shown as mean (SD) except total PSA (median with q25 and q75 values).

*Processed in Ln transformation.

^A,B,C^Results of pairwise comparisons among group means.

BMI: body index mass; NC: not Caucasian; N: no; Y:Yes; MetS: metabolic syndrome; Familial Hx: familial history.

tPSA: total prostate-specific antigen; fPSA: free prostate-specific antigen; LOOH: lipid hydroperoxide; RBC: red blood cell; AOPP: advanced oxidation protein products; PCB: protein carbonyl; -SH: thiol groups; TRAP: total radical-trapping antioxidant parameter.

**Table 2:** Results of multivariate Generalized Linear Model analysis examining the associations between diagnosis and biomarkers.

Tests	Dependent variable	Explanatory variables	F	df	p	Partial eta squared
Multivariate #1	tPSA _l_,f PSA, -SH, uric acid, AOPP, protein carbonyl, LOOH_plasma_, LOOH_RBC_	HC/BPH/PCa MetS Age BMI	16.99 2.62 2.48 2.61	16/358 8/179 8/179 8/179	<0.001 0.010 0.014 0.010	0.432 0.105 0.100 0.105
Between-subject effects	tPSA	HC/BPH/PCa	81.37	2/186	<0.001	0.467
	fPSA	HC/BPH/PCa	21.63	2/186	<0.001	0.189
	-SH	HC/BPH/PCa	55.69	2/186	<0.001	0.375
	AOPP	HC/BPH/PCa	11.53	2/186	<0.001	0.11
	PCB	HC/BPH/PCa	4.52	2/186	0.012	0.046
	LOOH_RBC_	HC/BPH/PCa	3.22	2/186	0.038	0.034
Multivariate #2	TRAP_plasma_, TRAP_RBC_	HC/BPH/PCa	2.41	4/288	0.049	0.032
Between-subject effects	TRAP_RBC_	HC/BPH/PCa	4.87	2/145	0.009	0.063

tPSA: total prostate-specific antigen; fPSA: free PSA; -SH: thiol group; AOPP: advanced oxidation protein products; PCB: protein carbonyl; LOOH: lipid hydroperoxide; RBC: red blood cell;

TRAP: total radical-trapping antioxidant parameter.

HC: healthy controls; BPH: benign prostatic hyperplasia; PCa: prostate cancer.

MetS: metabolic syndrome; BMI: body mass index.

**Table 3:** Model-generated estimated marginal means (SE) of the significant biomarkers as well as results of post-hoc comparisons in healthy controls (HC) and patients with benign prostate hyperplasia (BPH) and prostate cancer (PCa).

Variables	HC^A^	BPH^B^	PCa^C^
tPSA	−0.816 (0.096)^B,C^	−0.99 (0.087)^A,C^	+0.796 (0.080)^A,B^
fPSA	−0.568 (0.124)^B,C^	+0.055 (0.113)^A,C^	+0.536 (0.103)^A,B^
−SH groups	+0.598 (0.108)^C^	+0.339 (0.098)^C^	−0.732 (0.090)^A,B^
AOPP	−0.570 (0.137)^B,C^	+0.182 (0.125)^A^	+0.320 (0.114)^A^
PCB	−0.168 (0.147)^C^	−0.197 (0.133)^C^	+0.274 (0.122)^A,B^
LOOH_RBC_	−0.346 (0.148)^B,C^	+0.178 (0.135)^A^	−0.113 (0.124)^A^
TRAP_RBC_	+0.324 (0.145)^C^	−0.059 (0.156)	−0.362 (0.150)^A^

All values are shown as mean (SE) and as z scores.

^A,B,C^Results of pairwise comparisons among group means.

tPSA: total prostate-specific antigen; fPSA: free prostate-specific antigen; -SH: thiol group; AOPP: advanced oxidation protein products; PCB: protein carbonyl; LOOH: lipid hydroperoxide; RBC: red blood cell; TRAP: total radical-trapping antioxidant parameter.

**The Electronic Supplementary File (ESF)**, Table 1 shows the measurements of Hb and immune-inflammatory biomarkers namely hsCRP, ESR, leukocytes, and ferritin. However, no significant differences in any of those biomarkers could be found among the three study groups.

### Intercorrelation matrix between the variables

**Figure 1:**
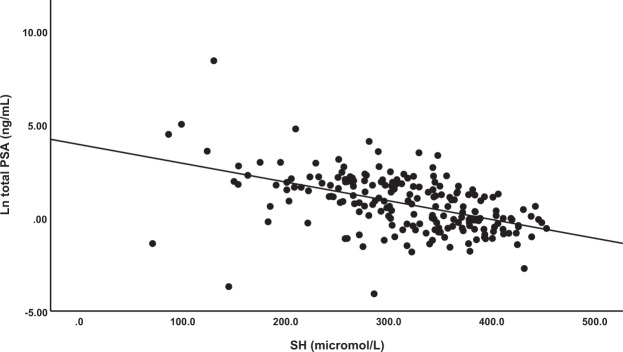
Correlation between total prostate-specific antigen (PSA) and thiol (−SH) groups.

The intercorrelation matrix between the biomarkers showed that tPSA was significantly associated with LOOH in red blood cells (LOOH_RBC)_ (r = 0.157, p = 0.028, n = 196), AOPP (r = 0.172, p = 0.014, n = 203), -SH groups (r = −0.527, p < 0.001, n = 204), and TRAP_RBC_ (r = −0.298, p < 0.001, n = 151). Figure 1 shows the strong association between tPSA and -SH groups (r = −0.527, p < 0.001, n = 204). There was a significant correlation between fPSA and tPSA (r = 0.825, p < 0.001, n = 204), -SH groups (r = −0.422, p < 0.001, n = 204) and TRAP_RBC_ (r = −0.243, p = 0.003, p = 151). Uric acid was significantly and positively correlated with TRAP in plasma (TRAP_plasma_) (r = 0.539, p < 0.001, n = 181), but less with TRAP_RBC_ (r = 0.177, p = 0.030, n = 151). AOPP was significantly correlated with PCB (r = 0.211, p = 0.003, n = 201). There was only a modest correlation between TRAP_plasma_ and RBCs (r = 0.200, p = 0.014, n = 151). tPSA and fPSA were not significantly associated with any of the immune-inflammatory variables (CRP, ferritin, ESR, WBCs). -SH groups were significantly associated with ESR (r = −0.279, p < 0.001, n = 194) and Hb (r = −0.334, p < 0.001, n = 195).

### Differences in biomarkers between PCa, BPH and HC

**Figure 2:**
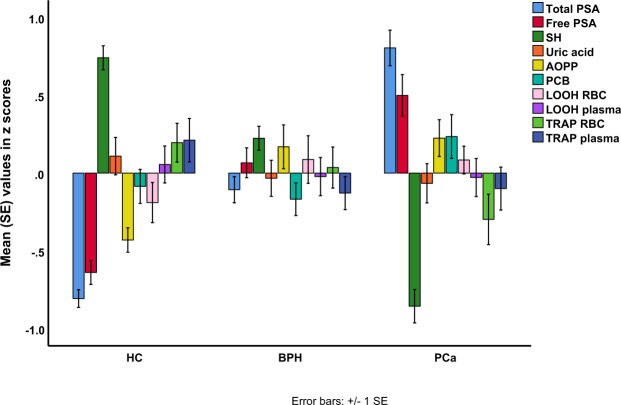
Total and free PSA levels and oxidative stress biomarkers (all in z values) in healthy controls (HC), patients with Benign Prostate Hyperplasia (BPH), and prostate cancer (PCa). PSA: prostate-specific antigen. -SH: thiol. AOPP: advanced oxidized protein products. PCB: protein carbonyls. LOOH RBC: lipid hydroperoxides in red blood cells. TRAP: total radical-trapping antioxidant parameter.

Figure 2 shows the values of PSA/OS biomarkers (all in z values) in HC, BPH and PCa. Table 2 shows the results of multivariate GLM analysis which examines the association between diagnosis and the PSA/OS biomarkers while adjusting for age, BMI and MetS. The TRAP data were assessed separately because TRAP was measured in a subset only (which would yield a considerable lowered number of dfs in the multivariate GLM analyses). Also, the immune-inflammatory markers were entered separately because they reflect another construct.

Multivariate GLM analysis #1 shows that diagnosis was significantly associated with PSA/OS biomarkers with huge effect sizes for tPSA (0.467) and -SH groups (0.375), moderate effect sizes for fPSA (0.189) and AOPP (0.110), and low impact sizes for PCB (0.046), TRAP_RBC_ (0.063) and LOOH_RBC_ (0.034). All differences remained significant after p-correction. There were no significant differences in LOOH_plasma_, uric acid and TRAP_plasma_ between the study groups.

Table 3 shows the model-generated estimated marginal means (in z scores) of the significant biomarkers as well as the results of post-hoc comparisons. tPSA and fPSA were significantly different between the three subgroups and increased from HC → BPH → PCa. -SH levels were significantly lower in PCa than in HC and BPH, whereas there were no significant differences between BPH and HC. AOPP was significantly increased in BPH and PCa as compared with HC, whereas PCB was higher in PCa than in HC and BPH. LOOH_RBC_ (but not plasma) was significantly higher in both BPH and PCa than in HC while TRAP_RBC_ (but not plasma) was significantly lower in PCa than in HC. There were no significant differences in the immune-inflammatory biomarkers CRP, WBCs, ESR, ferritin and Hb between the three study groups (F = 1.25, df = 10/362, p = 0.258) (see ESF Table 1). The ESF section 1 describes the possible effects of background variables (including the drug state) on the results.

### Prediction of PCa using the biomarkers

Table 4 shows the results of binary logistic regression analyses with PCa as dependent variable and either BPH or BPH + HC as reference group. Regression #1 shows that PCa (versus BPH + HC) was significantly predicted using tPSA, -SH groups and age as explanatory variables (X^2^ = 153.09, df = 3, p < 0.001, Nagelkerke = 0.724; 85.8% of all cases were correctly classified with a sensitivity of 79.5% and a specificity of 89.3%). Without age (regression #2) a similar prediction was established (X^2^ = 149.47, df = 2, p < 0.001, Nagelkerke = 0.713; whereby 87.3% of all cases were correctly classified with a sensitivity of 82.2% and a specificity of 90.1%).

**Table 4:** Results of binary logistic regression analysis with prostate cancer (PCa), metastasis and a suspicious digital rectal examination as dependent variables.

Regression	Dichotomy	Explanatory variables	Wald (df = 1)	p	OR	CI 95%
#1	PCa/HC + BPH	tPSA	29.07	<0.001	9.07	4.07–20.12
		-SH*	32.32	<0.001	9.82	4.47–21.59
		Age	3.4	0.065	0.94	0.88–1.004
#2	PCa/HC + BPH	tPSA	27.67	<0.001	7.15	3.44–14.88
		-SH*	29.77	<0.001	8.17	3.84–17.38
#3	PCa/HC + BPH	tPSA	22.13	<0.001	11.53	4.16–31.93
		-SH*	24.63	<0.001	10.17	4.07–25.42
		PCB	7.23	0.007	2.2	1.24–3.92
#4	PCa/BPH	tPSA	15.02	<0.001	8	2.80–22.87
		-SH*	20.36	<0.001	8.68	3.40–22.20
		PCB	7.31	0.007	2.22	1.25–3.95
#5	PCa/BPH + HC	Familial Hx	4.61	0.032	3.3	1.11–9.80
		Ethnicity	7.09	0.008	36.04	2.58–504.15
		PCB	4.87	0.027	1	1.07–3.03
		-SH*	26.71	<0.001	10.74	4.37–26.42
		tPSA	23.23	<0.001	7.62	3.36–17.40
#6	Metastasis/no	-SH	12.36	<0.001	0.12	0.04–0.39
#7	Suspicious rectal exam/normal rectal examination	-SH*	9.91	0.002	2.07	1.32–3.24
		tPSA	7.8	0.005	2	1.23–3.25
		AOPP	10.47	0.001	1.94	1.30–2.91
		MetS	5.14	0.023	0.37	0.16–0.87

HC: healthy control; BPH: benign prostatic hyperplasia; MetS: metabolic syndrome; OR: Odds ratio, CI: confidence intervals; tPSA: total prostate-specific antigen; AOPP: advanced oxidation protein products; -SH: thiol group; Familial Hx: familial history of PCa; -SH*: introduced as the inverse SH values.

Regression #3 shows a somewhat better prediction of PCa using tPSA, -SH and PCB (X^2^ = 144.89, df = 3, p < 0.001, Nagelkerke = 0.761, 91.1% of all cases were correctly classified with a sensitivity of 85.7% and a specificity of 94.0%). The same three variables yielded also a good prediction (regression #4) of PCa versus BPH (X^2^ = 87.62, df = 3, p < 0.001, Nagelkerke = 0.676, 87.1% of all cases were correctly classified with a sensitivity of 87.3% and a specificity of 86.9%). Regression #5 shows that a familial history of PCa, ethnicity, PCB, SH and tPSA significantly predicted PCa versus BPH + HC (X^2^ = 162.79, df = 5, p < 0.001, Nagelkerke = 0.762; 89.1% of all cases were correctly classified with a sensitivity of 81.7% and a specificity of 93.1%). Age was not significant in this regression (p = 0.093).

Table 5 shows the results of ROC analyses discriminating PCa from BPH + HC or PCa from BPH. The best separation was obtained for the combination of a familial history of PCa, ethnicity, PCB, -SH and tPSA (area under the ROC curve 0.950), followed by the combined effects of tPSA and -SH (0.945), and tPSA, -SH and PCB (0.945).

**Table 5:** Results of Receiver Operating Characteristics (ROC) analysis.

Dichotomy	Variables	ROC Area	SE	p-value	CI 95%
PCa/BPH + HC	tPSA	0.89	0.028	<0.001	0.836–0.944
	fPSA	0.754	0.037	<0.001	0.682–0.825
	−SΗ	0.881	0.025	<0.001	0.833–0.929
	tPSA + SH	0.945	0.014	<0.001	0.917–0.974
	tPSA + SH + PCB	0.945	0.014	<0.001	0.917–0.973
	FHx + Eth + PSA + SH + PCB	0.95	0.013	<0.001	0.929–0.982
PCa/BPH	tPSA_l_	0.838	0.035	<0.001	0.789–0.907
	fPSA	0.653	0.046	<0.001	0.562–0.743
	−SΗ	0.832	0.033	<0.001	0.766–0.897
	tPSA + SH	0.902	0.025	<0.001	0.853–0.950
	tPSA + SH + PCB	0.901	0.025	<0.001	0.852–0.949
	FHx + Eth + tPSA + SH + PCB	0.937	0.02	<0.001	0.898–0.975

PCa: Prostate cancer. HC: healthy control; BPH: benign prostate hyperplasia; tPSA: total prostate-specific antigen; fPSA: free prostate-specific antigen.

−SH: thiol group; PCB: protein carbonyl.

FHx: familial history; Eth: ethnicity.

Sums of biomarkers with or without demographic data (e.g. FA + Eth + PSA + SH + PCB): based on logistic regression analysis with 2, 3 or 5 explanatory variables.

### Associations among tPSA and -SH groups and other PCa features

**Figure 3:**
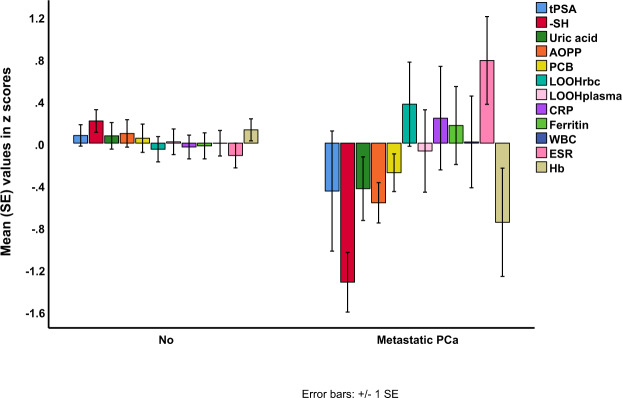
Differences in total PSA and oxidative stress biomarkers between patients with prostate cancer metastasis (metastatic PCa) versus patients without metastasis (No). PSA: prostate-specific antigen. SH: thiol. AOPP: advanced oxidized protein products. PCB: protein carbonyls. LOOH RBC: lipid hydroperoxides in red blood cells. CRP: high sensitivity C-reactive protein. WBC: white blood cells. ESR: erythrocyte sedimentation rate. Hb: hemoglobin.

Subsequently, we have examined the associations between the biomarkers and other features of PCa, including metastasis, rectal examination, AV groups (risk stratification according to NCCN Guidelines), prostate size and ultrasound. Figure 3 shows the biomarkers in PCa patients with and without metastasis. -SH groups are significantly lower in PCa patients with (mean z score = −2.07 ∀0.24) than without (mean = −0.60 ∀0.10) metastasis (F = 32.57, df = 1/68, p < 0.001, partial eta squared = 0.324), whereas there are no significant differences in any of the other OS biomarkers or tPSA (F = 2.33, df = 1/68, p = 0.131). Moreover, ESR is increased (F = 7.42, df = 1/65, p = 0.008) and Hb decreased (F = 6.72, df = 1/65, p = 0.012) in patients with metastatic PCa. Table 4, regression 6 shows that lowered -SH groups are significantly associated with metastatic PCa versus PCa without metastasis (X^2^ = 36.42, df = 1, p < 0.001, Nagelkerke = 0.471, and sensitivity of 50.0% and specificity of 98.4).

**Figure 4:**
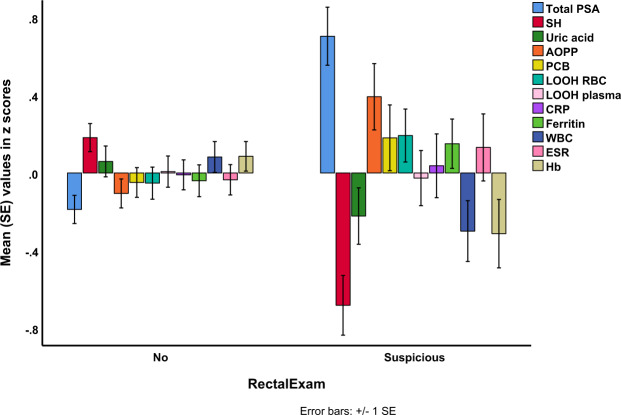
shows the biomarkers in those with a suspicious digital rectal examination versus a normal examination (See Fig. 3 for abbrevations).

Figure 4 shows the biomarkers in subjects (PCa, BPH and HC combined) examining a suspicious digital rectal examination versus a normal examination. tPSA (F = 30.71, df = 1/198, p < 0.001, partial eta squared = 0.134), -SH groups (F = 19.38, df = 1/198, p < 0.001, partial eta squared = 0.126) and AOPP (F = 13.59, df = 1/198, p < 0.001, partial eta squared = 0.065) were significantly associated with a suspicious digital rectal examination. Table 4, Regression #7 shows that -SH groups, tPSA, AOPP and MetS may predict a suspicious digital rectal examination (X^2^ = 49.56, df = 4, p < 0.001, Nagelkerke = 0.336). However, after bootstrapping (2000 bootstraps) tPSA was no longer significant (p = 0.06), while -SH groups, AOPP and MetS remained significant.

**Figure 5:**
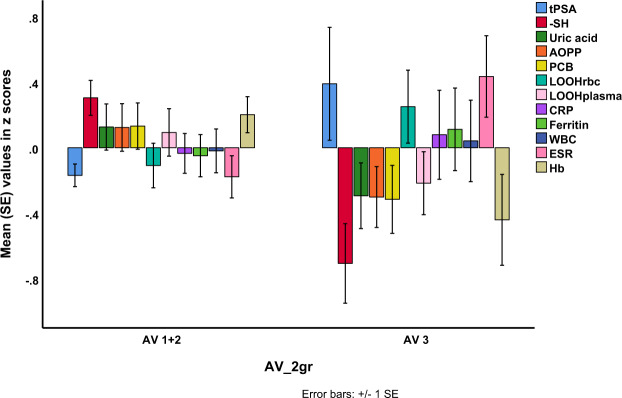
Shows that AV group 3 (risk group3) is characterized by lowered -SH group values (F = 8.91, df = 1/60, p = 0.004) and higher ESR (F = 9.03, df = 1/60, p = 0.004) than AV groups 1 + 2 (See Fig. 3 for abbreviations).

Figure 5 shows that the PCa AV risk group3 is accompanied by lower -SH levels as compared with PCa AV risk groups 1 + 2 (F = 16.41, df = 1/68, p < 0.001, partial eta squared = 0.194) while there were no significant differences in tPSA between those groups (F = 0.01, df = 1/68, p = 0.130, partial eta squared = 0.033). Moreover, patients in PCa AV risk group 3 showed lower Hb levels (F = 5.00, df = 1/65, p = 0.029) and higher ESR levels (F = 6.45, df = 1/65, p = 0.014) than patients in PCa AV risk groups 1 + 2.

Table 6 shows the results of multiple regression analysis predicting prostate volume (in cubic centimeter). The best prediction of prostate volume was obtained using age in combination with LOOH_RBC_ explaining 34.0% of the variance (Regression #1), whilst -SH levels were also significant and explained 9.1% of the variance (Regression #2). We found that 15.5% of the variance in ultrasound volume was explained by age and LOOH_plasma_ combined (Regression #3). Regression #4 shows that in PCa patients, tPSA was predicted by lower -SH levels, and increased PCB and prostate volume (27.5% of the variance). Regression #5 shows that in all participants combined, tPSA was significantly predicted by ethnicity, age and familial history of PCa (13.5% of the variance).

**Table 6:** Results of multiple regression analysis with prostate volume and ultrasound and total Prostate-Specific Antigen (PSA) as dependent variables.

Regression	Dependent variables	Explanatory variables	F	p	F model	df	p	Partial Eta Squared
#1	Prostate Volume	Age	+8.65	<0.001	39.60	2/154	<0.001	0.340
		LOOH_RBC_	+2.27	0.024				
#2	Prostate Volume	−SH	−3.93	<0.001	15.42	1/155	<0.001	0.091
#3	Ultrasound*	Age	+3.35	0.001	9.72	2/106	<0.001	0.155
		LOOH_plasma_	+2.50	0.014				
#4	tPSA	−SH	−5.00	<0.001	21.85	3/173	<0.001	0.275
		PCB	+2.01	0.046				
		Prostate size	+4.22	<0.001				
#5	tPSA	Ethnicity	+2.85	0.005	10.41	3/200	<0.001	0.135
		Age	+4.34	<0.001				
		Familial Hx	+2.14	0.034				

tPSA: total prostate-specific antigen; LOOH: Lipid hydroperoxide; RBC: red blood cells; -SH: thiol group.

PCB: protein carbonyl; Familial Hx: familial history of PCa.

## Discussion

The main findings of this study are: (a) PCa is characterized by lowered plasma -SH groups and red blood cell TRAP levels and higher plasma AOPP and PCB levels as compared with BPH and control subjects, and (b) OS biomarkers may be used together with tPSA as external validating criterion for PCa. Importantly, 10-fold cross-validation showed that tPSA and fPSA values together with -SH, PCB and AOPP yielded a validation accuracy of 96.34% when differentiating PCa from BPH and HC. Lowered -SH levels show an adequate diagnostic performance for PCa with an AUC ROC of 0.881 (versus controls and BPH) while lowered -SH groups combined with higher tPSA values yielded an area under the ROC curve of 0.945.

### Antioxidant Defenses in PCa and BPH

SH-disulphide homeostasis is crucial in several processes such as antioxidant defense, detoxification, cell signaling, transcription, protein regulation and apoptosis^32,33^. Previous studies reported decreased -SH groups in advanced non-small cell lung cancer^34^ and advanced gastric adenocarcinoma^35^. However, a previous study found an increase of non-protein -SH levels (GSH, Reduced Glutathione) in plasma and erythrocytes in PCa patients, mainly in presence of bone metastasis. The authors suggested that the increased levels could be a compensatory mechanism to prevent tissue damage caused by oxidative stress^36^. In plasma, protein -SH levels are more abundant than glutathione, representing approximately 70% of total intracellular pool of reduced -SH. However, GSH levels in plasma are very low and do not reflect all -SH groups^37^. Interestingly, in our study there was a strong association between total PSA and -SH levels. One hypothesis is that increased PSA peptidase activity could have induced lowered -SH levels. However, since PSA activity involves serine proteinase and not cysteinases this is less plausible^38,39^.

Some studies reported decreased antioxidant defenses, other than -SH groups, in PCa and BPH^19^ including superoxide dismutase (SOD) and catalase in patients with PCa metastasis^36,40^. However, few reports measured total antioxidant capacity, which reflects the cumulative effects of different antioxidant defenses in plasma thereby providing a more integrated parameter of antioxidant defenses^41^. In this respect, we found that TRAP in erythrocytes, but not plasma, was decreased in patients with PCa as compared with HC. A previous study demonstrated that antioxidant status, evaluated by TEAC (Trolox Equivalent Antioxidant Capacity), was reduced in plasma of PCa patients when compared with BPH and HC^42^. Pande *et al*.^13^ reported that patients with advanced stages of PCa had lower TEAC, suggesting increased antioxidant consumption.

### Lipid and protein oxidation in PCa and BPH

The results of the present study did not show a significant increase in the lipoperoxidation marker LOOH either in plasma or red blood cells. Our data are in agreement with a previous study, which showed no difference in lipoperoxidation between PCa and BPH^43^. Previous studies on aldehyde formation (which is the consequence of lipid peroxidation) in prostate disease including TBARS (Thiobarbituric Acid Reactive Substances) and MDA yielded controversial results. TBARS in erythrocyte lysates was increased in BPH patients when compared with HC^42,44^ while MDA levels were higher in BPH patients than HC^17,40,45,46^ while there was a strong correlation between MDA and tPSA^17^. However, other studies found no differences in MDA levels in BPH patients as compared with controls^47^.

Our results showed increased levels of the protein oxidation indices in PCa namely increased PCB in PCa patients versus BPH, and increased AOPP in patients with PCa and BPH versus controls. PCB may be formed by the oxidation of a few amino acid side chains via the addition of aldehydes such as those generated from lipid peroxidation. PCB is an initial and reversible product from protein oxidation whilst AOPP is the final and irreversible product of protein oxidation. Pande *et al*.^13^ showed that PCB levels were higher in PCa patients as compared to HC although another case-control study reported no significant associations between PCa risk or aggressiveness and PCB^48^.

Prostatic inflammation as a result of infection, urine reflux, hormonal and immune dysbalances^11,49^ may be involved in the pathogenesis and progression of PCa by causing cell and DNA damage and promoting cellular turnover. Prostate tissue damage and oxidative stress generated from inflammation could lead to compensatory cellular proliferation with the resulting hyperplastic growth^50^. Nevertheless, in the present study, we did not find significant associations between oxidative stress biomarkers and inflammatory biomarkers including ferritin, ESR, and hsCRP. Moreover, these inflammatory biomarkers were not altered in PCa or BPH, suggesting that oxidative stress toxicity may be involved in the physiopathology of PCa independently of immune-inflammatory processes. As such, increased OS and lowered antioxidant defenses, including lowered -SH groups and TRAP as well as protein oxidation may be more important than immune-inflammatory processes in the pathophysiology of PCa.

### Oxidative stress and staging of PCa

Interestingly, our study found that there was a significant association between a suspicious rectal examination and lowered levels of -SH groups and AOPP, whereas the association with tPSA was no longer valid after bootstrapping. Also, these findings suggest a pathogenic role of -SH groups and AOPP in PCa. As such, our algorithm comprising -SH groups and tPSA may be used to differentiate BPH from PCa in subjects with suspicious digital rectal exam. Likewise, prostate volume was predicted by pre-surgery values of -SH groups and LOOH in red blood cells, but not tPSA. In this respect, increased levels of tPSA were significantly predicted by lowered -SH groups, increased PCB and prostate volume, indicating that -SH groups and OS processes are involved in BPH, cancer development and increased production of tPSA. Moreover, our results show that lowered -SH groups and Hb, and increased ESR, but not PSA, predict a high-risk phenotype as indicated by the association of -SH with metastatic PCa and AV risk group 3. Previously, it was shown that progressive stages in PCa are associated with a more detrimental redox status^13^. In addition, it was reported that increased ROS levels are closely linked to the accelerated formation of metastasis^51^.

### Limitations of the study

In the present study, BPH and PCa patients were somewhat older and they showed a higher frequency of MetS, factors that could have influenced the results. Nevertheless, our data were statistically adjusted for possible effects of these and other extraneous variables. Age, BMI and MetS had moderate effects (effect sizes around 0.10) on the biomarkers, whilst diagnosis yielded a huge effect size (0.432). The presence of a greater number of patients with MetS in the BPH group is consistent with findings in the literature that reported associations between the pathophysiology of metabolic imbalance and the predisposition of BPH. However Gacci *et al*.^10^ in a meta-analysis comprising 24 studies including 132.589 participants concluded that patients with PCa and MetS have a worse prognosis and a more aggressive phenotype of PCa. This study would have been even more interesting if we had measured biomarkers of oxidative DNA damage. Since antioxidant depletion results in oxidative stress toxicity including DNA damage, oxidative DNA biomarkers could contribute to the diagnostic performance established here. In PCa patients, there were more non-Caucasians as compared with the BPH study group while the levels of -SH groups were lower in Black people than in Caucasians and Asians combined. Therefore, future research should examine whether lowered -SH groups in Black men may explain that the latter are at higher risk of PCa than White and Asian men. Future research should examine whether levels of -SH groups may be used as an adjunctive method for follow up of patients on active surveillance.

## Conclusions

This study showed a robust association between -SH groups, OS biomarkers and PCa and its prognosis. -SH levels combined with tPSA may be used as an external validating criterion for PCa and to differentiate PCa from BPH in patients with suspicious digital rectal exam. This is the first study to propose models using oxidative stress biomarkers and clinical and laboratory data to predict prognosis in PCa. If other reports confirm our results, new drugs could be tried with -SH as targets.

## Supplementary information

Supplementary information
