# Supplementary material for: Thiol Groups as a Biomarker for the Diagnosis and Prognosis of Prostate Cancer
Source: Sci Rep. 2020 Jun 4;10:9093. doi: 10.1038/s41598-020-65918-w (PMC7272452; doi:10.1038/s41598-020-65918-w)
Supplement: Supplementary file 1 — Supplementary information [file 41598_2020_65918_MOESM1_ESM.docx]

**ELECTRONIC SUPPLEMETARY FILE (ESF)**

**Thiol Groups as a Biomarker for the Diagnosis and Prognosis of Prostate Cancer**

Alexsandro Koike^1^, Brunna Emanuella França Robles^2^, Ana Gabriela da Silva Bonacini^3^, Camila Cataldi de Alcantara^3^ Edna Maria Vossoci Reiche^4^, Isaias Dichi^5^, Michael Maes^6^, Rubens Cecchini^7^, Andréa Name Colado Simão^4^

1. Cancer Institute of Londrina, Laboratory of Research in Applied Immunology, University of Londrina, Londrina, Paraná, Brazil

2. Laboratory of Research in Applied Immunology, University of Londrina, Londrina, Paraná, Brazil

3. Laboratory of Research in Applied Immunology, University of Londrina, Londrina, Paraná, Brazil

4. Department of Pathology, Clinical Analysis and Toxicology, Laboratory of Research in Applied Immunology, University of Londrina, Londrina, Paraná, Brazil

5. Department of Internal Medicine, University of Londrina, Londrina, Paraná,

6. IMPACT Strategic Research Centre, School of Medicine, Deakin University, Geelong, VIC, Australia.

7. Department of Pathology Sciences, University of Londrina, Londrina, Paraná, Brazil

Corresponding author: Andréa Name Colado Simão

Postal address: Department of Patology, Clinical Analisys and Toxicology – Avenida

Robert Koch, n.60. University of Londrina, Londrina, Paraná, Brazil. CEP 86038-440.

Tel.: +55-43-3371-2321; Fax +55-43-3371-2619. E-mail address: [deianame@yahoo.com.br](mailto:deianame@yahoo.com.br)

**ESF Table 1.** Immune-inflammatory biomarkers in healthy controls (HC), and patients with prostate hyperplasia (BPH) and prostate cancer (PCa)

| **VARIABLES** | **HC** | **BPH** | **PCa ^C^** | **F** | **df** | **p** |
| --- | --- | --- | --- | --- | --- | --- |
| hsCRP (mg/L) | 5.44 (8.57) | 3.26 (4.47) | 5.40 (18.60) | 1.28 | 2/201 | 0.280 |
| Ferritin (μg/L) | 293.1 (229.3) | 275.3 (201.1) | 281.1 (262.9) | 0.00 | 2/201 | 1.000 |
| Hb (g/dL) | 15.52 (1.41) | 15.33 (1.23) | 14.86 (1.45) | 17.71 | 2 | <0.001 |
| Leukocytes (uL) | 6.72 (2.02) | 6.87 (1.69) | 6.50 (1.88) | 1.05 | 2/192 | 0.353 |
| ESR (mm/hour) | 12.47 (11.15) | 13.86 (12.04) | 16.17 (16.91) | 1.04 | 2/191 | 0.356 |

All values are shown as mean (SD)

Data are processed in Ln transformation

hsCRP: high-sensitive C-Reactive Protein

Hb: Hemoglobin

ESR: Erythrocyte Sedimentation Rate

**ESF section 1: Effects of background variables**

Age is significantly associated with tPSA (r=0.311, p<0.001, n=204), fPSA (r=0.328, p<0.001, n=204) and -SH groups (r=-0.402, p<0.001, n=204). BMI is significantly associated with tPSA (r=-0.158, p=0.024, n=203), fPSA (r=-0.177, p=0.011, n=203), LOOH_plasma_ (r=-0.227, p=0.001, n=203), and uric acid (r=0.224, p=0.001, n=203). ESR was significantly associated with age (r=0.174, p=0.016, n=194) and BMI (r=0.195, p=0.004, p=193) (data not shown).

Multivariate regression #1 (Table 2) shows that not only age, but also MetS and BMI had a significant impact on tPSA and OS biomarkers. AOPP was significantly higher in subjects with MetS than in those without MetS (F=15.93, df=1/186, p<0.001). Age was significantly associated with -SH groups (F=10.02, df=1/186, p=0.002, R^2^=0.051) and AOPP (F=5.73, df=1/186, p=0.018, R^2^=0.030), while BMI was correlated with uric acid (F=8.46, df=1/186, p=0.004, R^2^=0.043). We found no significant effects of familial history of PCa on the dependent variables listed in table 2 (F=1.57, df=1/180, p=0.136). There was a significant effect of ethnicity on -SH groups (F=5.46, df=1/187, p=0.020) with lower -SH levels in black people (mean in z-values=-0.389, SE=0.193) than in Caucasians + Asians (mean=+0.090, SE=0.069) (data not shown).

We have also examined possible effects of the drug state of the patients; 19 subjects were treated with doxazosine, 7 with finasteride, 84 with antihypertensive drugs, 30 with hypercholesterolemia drugs, 23 with hypoglycemic drugs and 16 with non-steroidal anti-inflammatory drugs (NSAIDs). Introducing these drug state variables in Regression #1 showed that doxazosine (F=0.77, df=8/173, p=0.629), finasteride (F=0.19, df=8/173, p=0.992), antihypertensive drugs (F=0.28, df=8/173, p=0.972), hypercholesterolemia drugs (F=0.72, df=8/173, p=0.68), hypoglycemic drugs (F=1.68, df=8/173, p=0.106) and NSAIDs (F=1.52, df=8/173, p=0.153) had no significant effects, while the effects of diagnosis remained significant (F=14.89, df=16/348, p<0.001). In addition, all tests of between-subject effects of all drug state variables on tPSA / OS biomarkers were non-significant, except a positive association (without p-correction) between NSAIDs and fPSA (F=4.71, df=1/, p=0.031). There were no significant effects of the drug state variables on both TRAP values and the immune-inflammatory variables. Finally, there were no significant effects of smoking and diabetes on any of the biomarkers examined (data not shown).
